# Trastuzumab Mechanism of Action; 20 Years of Research to Unravel a Dilemma

**DOI:** 10.3390/cancers13143540

**Published:** 2021-07-15

**Authors:** Hamid Maadi, Mohammad Hasan Soheilifar, Won-Shik Choi, Abdolvahab Moshtaghian, Zhixiang Wang

**Affiliations:** 1 Department of Oncology, Cross Cancer Institute, University of Alberta, Edmonton, AB T6G 1Z2, Canada; hmaadi@ualberta.ca (H.M.); wonshik@ualberta.ca (W.-S.C.); 2 Department of Medical Laser, Medical Laser Research Center, Yara Institute, ACECR, Tehran 1315795613, Iran; h.soheilifar@edu.umsha.ac.ir; 3 Department of Molecular and Cell Biology, Faculty of Basic Sciences, University of Mazandaran, Babolsar 4741695447, Iran; vahabmoshtagh@semums.ac.ir; 4 Deputy of Research and Technology, Semnan University of Medical Sciences, Semnan 3514799442, Iran; 5 Department of Medical Genetics and Signal, Transduction Research Group, Faculty of Medicine and Dentistry, University of Alberta, Edmonton, AB T6G 2H7, Canada

**Keywords:** trastuzumab, breast cancer, HER2, PI3K/AKT pathway, MAPK pathway

## Abstract

**Simple Summary:**

Overexpression of HER2 receptors have been identified in various types of cancer including breast cancer and ovarian cancer. HER2 overexpression is generally associated with poor clinical outcomes in patients with HER2-positve tumors. Trastuzumab, an antibody specifically targeting HER2 receptors, showed promising clinical benefits for patients with HER2-positive tumors. Studies show that trastuzumab suppresses HER2 receptors’ oncogenic functions in HER2-postive tumors. Moreover, trastuzumab has been shown to provoke immune responses against the HER2-amplified tumors.

**Abstract:**

Trastuzumab as a first HER2-targeted therapy for the treatment of HER2-positive breast cancer patients was introduced in 1998. Although trastuzumab has opened a new avenue to treat patients with HER2-positive breast cancer and other types of cancer, some patients are not responsive or become resistant to this treatment. So far, several mechanisms have been suggested for the mode of action of trastuzumab; however, the findings regarding these mechanisms are controversial. In this review, we aimed to provide a detailed insight into the various mechanisms of action of trastuzumab.

## 1. Introduction

HER2 overexpression in approximately 20–30% of breast cancer (BC) patients has motivated researchers for years to find a decent drug to specifically target HER2. A humanized monoclonal antibody named trastuzumab (Herceptin^®^), which targets the extracellular domain of HER2, was developed. Since the approval of trastuzumab, many investigations have been carried out to unravel the trastuzumab mechanism of action and find an appropriate combination therapy to enhance the treatment efficacy. In this review, we aimed to shine the light on the various mechanisms through which trastuzumab exerts its anti-tumoral effects.

## 2. From Characterization of HER2 to Developing Trastuzumab

**Figure 1:**
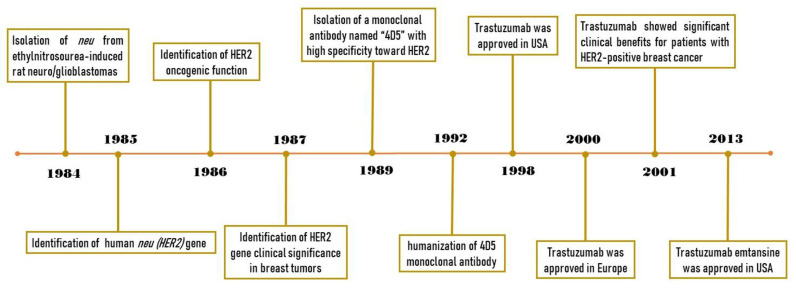
Timeline of trastuzumab history, from HER2 identification to demonstrating the clinical benefits of trastuzumab.

In 1984, Schechter et al. isolated a gene from ethylnitrosourea-induced rat neuro/glioblastomas and confirmed the ability of this gene to transform the NIH3T3 cells [1]. This gene, which is named ‘*neu*’, was later shown to encode a protein with 185 kDa molecular weight [1]. One year later, Coussens et al. identified and characterized a gene located at the human q21 region of chromosome 17 which was homologous to the rat *neu* gene [2]. Due to the significant similarities in the protein sequence between the EGFR (HER1) and the product of the *neu* gene, it was named HER2 and suggested to play a role as a kinase receptor at the cell surface. In 1986, Drebin et al. demonstrated the tumorigenic potential of NIH3T3 cells, when transfected with the *HER2* gene in vivo [3]. They also showed that treating the mice with an antibody against the *neu* gene product (anti-HER2 antibody) significantly suppresses tumor growth. The oncogenic function of HER2 was also confirmed by another research group about a year later [4]. In 1987, Slamon et al. found that the HER2 gene is amplified in about 30% of BC tumors, and this amplification is significantly correlated with poor prognosis in BC patients [5]. In 1989, Hudziak et al. isolated a monoclonal antibody named “4D5”, which has a high specificity toward HER2, from mice exposed to this receptor and showed its antiproliferative effects against SKBR3 HER2-positive BC cells [6]. In 1992, Carter et al. humanized the 4D5 monoclonal antibody which made it suitable for further clinical evaluation [7]. This antibody, which was named “Trastuzumab”, showed significant clinical benefits toward patients with HER2-positive BC and increased the efficacy of conventional chemotherapies [8]. Finally, trastuzumab was approved in the USA and Europe in 1998 and 2000, respectively [9,10]. The key findings related to trastuzumab history are summarized in Figure 1. After demonstrating the clinical benefits of trastuzumab, many studies were carried out in an attempt to unravel the mechanisms of action of trastuzumab. In this review, we discuss different mechanisms of action proposed for trastuzumab.

## 3. HER2 Signaling

The ErbB receptor tyrosine kinase 2 (ErbB2 or HER2) belongs to the ErbB (HER) family of receptor tyrosine kinases along with three other members, including epithelial growth factor receptor (EGFR), ErbB3 (HER3), and ErbB4 (HER4) [11]. Generally, these receptors consist of three main domains: (1) the extracellular domain that contains the four subdomains that mediate HER receptor ligand-dependent or -independent dimerization, (2) transmembrane domain that connects extracellular and cytoplasmic domains, and (3) the C-terminal cytoplasmic domain that contains tyrosine kinase and regulatory subdomains [12]. Upon the binding of HER-specific ligands to HER receptors, these receptors form homo- or heterodimers. Subsequently, certain tyrosine residues in cytoplasmic domains are auto-phosphorylated or trans-phosphorylated to activate several intracellular signaling pathways [13,14]. Structural analysis revealed an open conformation for HER2 extracellular domain similar to a ligand-activated state of other HER receptors, suggesting that these receptors form homo- and heterodimers independent of ligands [15]. Until now, no HER2-specific ligand has been identified, consistent with what was found in the structural analysis. Therefore, HER2 is a preferred binding partner in HER receptors’ dimerization. In pathological conditions, the overexpression of HER2 facilitates the ligand-independent HER2 homo- and heterodimerization and activates multiple downstream signaling pathways involved in the malignant properties of cancer cells [16]. Therefore, the inhibition of HER2 dimerization plays an important role in the suppression of HER2-mediated cell signaling and tumor growth.

**Figure 2:**
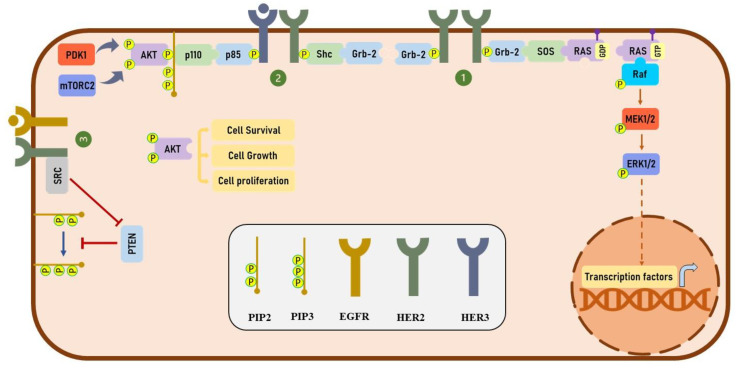
HER2-mediated downstream signaling pathway. (**1**) HER2-mediated activation of MAPK signaling pathway. Upon homodimerization of HER2 receptors, these receptors are phosphorylated at different phosphorylation sites. Phosphorylation of HER2 provides binding sites for Grb-2 proteins which bind to SOS with GEF activity. SOS then activates RAS by replacing the GDP with GTP. The activated RAS phosphorylates Raf, and Raf phosphorylates MEK. Finally, ERK is phosphorylated and activated by MEK which enables this protein to enter the nucleus and activate the transcription of genes involved in various cellular processes. (**2**) HER2-mediated activation of PI3K/AKT signaling pathway: upon the binding of HER3-specific ligands, such as heregulin (HRG) to HER3, the HER2-HER3 heterodimers are formed and HER2 phosphorylates HER3 at different tyrosine residues. The phosphorylated HER3 provides docking sites for p85 subunit of PI3K. The fully activated PI3K complex is generated by binding of p110 subunit of PI3K to p85 which is able to produce PIP3 via phosphorylating the PIP2. AKT binds to PIP3 and is phosphorylated by PDK1 and mTORC2. Phosphorylation of AKT at both threonine and serin phosphorylation sites significantly increases its catalytic activity which regulates the function of various proteins involved in several cellular processes. (**3**) HER2-mediated inhibition of PTEN activity: SRC binds to HER2 receptors and inhibits the activity of PTEN via mediating the phosphorylation of this enzyme. As a phosphatase, PTEN dephosphorylates PIP3 and therefore inhibits the binding of AKT to PIP3.

Phosphorylation of HER2 at specific phosphorylation residues provides docking sites for several downstream effectors, including growth factor receptor-bound protein 2 (Grb2) and Src homology 2 domain containing transforming protein (Shc), which play an important role in regulating the mitogen-activated protein kinase (MAPK) signaling pathway [17,18]. HER2 has been shown to activate the phosphatidylinositol 3-kinase (PI3K)/protein kinase B (PKB/AKT) signaling pathway both directly and indirectly. Heterodimerization of HER2 with HER3 activates the PI3K/Akt signaling pathway through the phosphorylation of HER3 receptors at multiple tyrosine residues [19]. It has been shown that the phosphorylation of HER3 provides several binding sites for a p85 subunit of PI3K and significantly activates the PI3K/AKT signaling pathway [19,20]. Furthermore, HER2 has been shown to indirectly activate the PI3K/AKT signaling through Src-mediated inhibition of phosphatase and tensin homolog (PTEN) activity [21]. On the other hand, Ruiz-Saenz et al. have revealed that the p85 regulatory subunit of PI3K directly binds to HER2 at tyrosine 1139, which suggest that HER2 directly activates the PI3K/AKT pathway [22]. Altogether, in various types of cancers, HER2 plays a critical role in activating MAPK and PI3K/Akt signaling pathways. The detail of these signaling pathways is illustrated in Figure 2.

## 4. Trastuzumab and HER2 Dimerization

Trastuzumab has been shown to bind three distinct regions of domain IV of the HER2 extracellular domain through electrostatic and hydrophobic bindings [15,23]. Initially, it was speculated that trastuzumab inhibits tumor growth through blocking HER2 dimerization with other HER receptors. However, studies reported controversial results regarding the effects of trastuzumab on HER2 homo- and heterodimerization. Generally, the effects of trastuzumab on HER2 dimerization are evaluated in two different conditions: (1) the effect of trastuzumab on ligand-dependent HER2 heterodimerization, and (2) the effect of trastuzumab on ligand-independent HER2 homo- and heterodimerization. In the presence of HER-specific ligands, trastuzumab has no significant effects on HER2 heterodimerization [16,23,24]. On the other hand, findings about the effect of trastuzumab on ligand-independent HER2 heterodimerization are remained controversial. For instance, Gaborit et al. demonstrated that trastuzumab significantly blocks EGFR-HER2 heterodimerization in the SKOV-3 HER2-positive ovarian cancer cell line using a Fluorescence Resonance Energy Transfer (FRET)-based method [25]. On the contrary, Diermeier et al. showed that trastuzumab is unable to suppress the EGFR-HER2 heterodimerization in HER2-positive BC cell lines, BT474 and SKBR3, using a similar approach [16]. Using a reversible cross-linker, Junttila et al. showed that trastuzumab disrupts ligand-independent heterodimer formation while ligand-dependent heterodimerization remains unaffected [24]. To assess the effect of trastuzumab on HER2 homodimerization, we used the Chinese hamster ovary (CHO) cells overexpressing human HER2 [26]. CHO cells have been shown to have little to no expression of all HER receptors, making it a good model to study homodimerization [27,28]. Surprisingly, our results showed that trastuzumab induces HER2 homodimerization in a dose- and time-dependent manner [26]. These results are consistent with previous reports showing enhanced HER2 homodimerization upon trastuzumab treatment in HER2-positive BC cell lines, SKBR3 and BT474 [16]. It is yet to be determined how trastuzumab concurrently induces HER2 homodimerization and inhibits ligand-independent HER2 heterodimerization. Taken together, these findings suggest that trastuzumab has limited effects on HER2 homo- and heterodimerization.

## 5. Trastuzumab and HER Phosphorylation

Using the CHO cells overexpressing EGFR, HER2, or HER3, we showed that trastuzumab specifically binds to HER2 and does not bind to EGFR and HER3 [26]. Therefore, trastuzumab potentially affects the HER2-mediated signaling directly and other members of HER family-mediated signaling indirectly. Contrary to the initial expectation of a possible inhibitory role of trastuzumab on HER2 phosphorylation, several studies have shown that trastuzumab induces HER2 phosphorylation [16,29,30,31,32]. So far, three mechanisms have been suggested for increasing the phosphorylation of HER2 by trastuzumab. First, as discussed, trastuzumab increases the HER2 homodimerization [26]. Second, trastuzumab induces tyrosine kinase activity of HER2, and hence increases the phosphorylation of HER2 upon homodimerization [29]. Third, trastuzumab increases the HERs-specific ligand production to activate other HER receptors through the upregulation of ADAM17 [32]. ADAM proteases play a significant role in generating HER ligands from pro-HER ligands [33]. Increased HERs-specific ligand production leads to ligand-dependent HER2 heterodimerization, thereby inducing HER2 phosphorylation [32]. However, as discussed earlier, the majority of studies found that trastuzumab had little to no effect on ligand-dependent HER2 heterodimerization and phosphorylation. It is worth mentioning that higher phosphorylation of HER2 at tyrosine 1248 is positively correlated with good prognosis in patients with HER2-positive BCs upon trastuzumab therapy [29]. It is yet to be exactly determined how trastuzumab concurrently induces HER2 phosphorylation and inhibits cancerous features of HER2-positive BC cells.

Although the effect of trastuzumab on EGFR and HER4 phosphorylation is not well-studied, evidence supporting the inhibitory role of trastuzumab on HER3 phosphorylation is quite robust [24,29,32]. Moreover, it has been shown that trastuzumab can induce HER3 degradation [29]. Altogether, current findings suggest that trastuzumab increases HER2 phosphorylation while suppressing HER3 phosphorylation.

## 6. Trastuzumab and HER2 Endocytosis and Degradation

The effects of trastuzumab on inducing the HER2 endocytosis and degradation are controversial. Although some studies show the downregulation of HER2 upon trastuzumab treatment [32,34,35,36,37], others show no effect [31,38,39]. Generally, studies have shown that the long-term treatment of trastuzumab downregulates HER2 but did not provide a clear mechanism on how trastuzumab downregulates HER2. Of note, a few studies have shown that trastuzumab decreases the expression of other HER receptors including HER3 and EGFR [29,35,36].

The effect of trastuzumab on HER2 endocytosis has been well studied by Austin et al. [38]. They showed that HER2 receptors are frequently internalized and recycled to the plasma membrane and trastuzumab had no effects on this cycle. Their extensive results have revealed that, although trastuzumab is detected in internalized vesicles a few hours after the treatment of the BC cells, these vesicles are mainly recycling endosomes rather than degrading ones. The majority of trastuzumab, along with HER2 receptors, was recycled to the plasma membrane in a short period of time. Using the same BC cell lines, Dokmanovic et al. detected trastuzumab internalization and HER2 degradation after treating the cells for four days [34]. Intriguingly, the localization of internalized trastuzumab bound to HER2 in early endosomes was shown in both studies. In addition, in vitro experiments unraveled the negative correlation between caveolin-1 (CAV1) and HER2 expression [40]. It has been shown that the suppression of CAV1-mediated HER2 endocytosis stabilizes HER2 at cell membrane and increases HER2-trastuzumab complex formation in HER2-positive tumors, which ultimately enhances trastuzumab-induced antibody-dependent cell-mediated cytotoxicity (ADCC) [40]. Furthermore, Scaltriti et al. showed that HER2 ubiquitination and degradation can be elevated after trastuzumab treatment [37]. Taken together, our knowledge about the possible role of trastuzumab on HER2 endocytosis is limited. More studies should be carried out to unravel the molecular mechanism(s) through which trastuzumab modulates HER2 endocytosis and degradation.

## 7. Trastuzumab and the MAPK Signaling Pathway

The MAPK pathway, one of the well-studied signaling pathways, plays an important role in various cellular processes, such as cell proliferation, differentiation, apoptosis, migration, and stress responses [41,42]. In addition, this pathway is highly activated in different types of cancer [43,44]. Generally, four different MAPK downstream effectors, including extracellular signal-regulated kinase1/2 (ERK1/2), ERK5, p38 MAPK, and c-JUN N-terminal kinase (JNK), are activated in response to different stimuli [42]. The activation of RTKs by extracellular ligands such as epidermal growth factor (EGF) activates the ERK1/2 and ERK5; however, two other effectors (p38 MAPK and JNK) are generally activated by cellular and environmental stresses [45,46]. Moreover, it has been shown that leukemia inhibitory factor (LIF) and the signal transducer and activator of transcription-3 (STAT-3) activates mitogen-activated protein kinase 5 (MEK5), which subsequently activates ERK5 in normal and cancer cells [47,48,49]. In the EGFR signaling pathway, the binding of HER-specific ligands to HERs induce receptors’ homo- and heterodimerization and phosphorylation [13,14]. Phosphorylation of HERs at specific residues provides docking sites for various adaptor proteins including Grb-2 [17,18]. Grb-2 binds to a protein with guanine nucleotide exchange factor (GEF) activity named Son of Sevenless (SOS), which in turn activates the Rat Sarcoma viral proto-oncogene (RAS) protein by replacing the guanosine triphosphate (GTP) with guanosine diphosphate (GDP) [41,50]. The activation of RAS mediates the activation and phosphorylation of a cascade of downstream serine/threonine kinases, which ultimately ends with the phosphorylation of ERK1/2 protein [50]. The phosphorylated and dimerized ERK1/2 proteins are translocated into the nucleus to regulate the function of transcription factors playing pivotal roles in various cellular processes [51]. In HER2-positive BC, due to the overexpression of HER2 receptors, the formation of HER2 homodimers, and therefore HER2 phosphorylation, are significantly elevated. Increased phosphorylation of HER2 receptors provides several binding sites for different adaptor proteins involved in regulation of MAPK signaling pathway [17,18], and hence hyperactivates this signaling pathway in HER2-positive BC tumors. Therefore, trastuzumab potentially affects the MAPK pathway through intervening with the HER2 receptors’ normal functions.

Different studies have suggested both inhibitory and activatory roles of trastuzumab on MAPK signaling. However, the experimental conditions are not consistent in these studies. ERK1/2 phosphorylation was increased when cells are exposed to short term treatment of trastuzumab [29,31,32]. For instance, Bagnato et al. have shown that treatment of SKBR3 HER2-positive BC cells with trastuzumab for 2 minutes significantly increases ERK1/2 phosphorylation [31]. Although several studies have indicated that trastuzumab inhibits the MAPK signaling pathway [23,52,53,54,55,56,57], the findings suggest that the effect of trastuzumab on MAPK signaling can be altered by some conditions. Watanabe et al. have demonstrated that, even though trastuzumab inhibits ERK1/2 phosphorylation in SKBR3 HER2-positive BC cells, it does not have any effect on ERK1/2 phosphorylation in BT474 HER2-positive BC cells [58], suggesting the cell line-specific effects of trastuzumab on MAPK signaling. Using MCF10A human breast epithelial cells stably transfected with a plasmid containing chimeric human HER2 and a FK506-binding protein (FKBP), Ghosh et al. tested MAPK activation when cells are cultured with three different molecules: (1) transforming growth factor α (TGFα) for HER2-EGFR heterodimerization, (2) heregulin for HER2-HER3 heterodimers, and (3) AP1510, an artificial ligand to homodimerize HER2-FKBP proteins [54]. They showed that trastuzumab induces disassociation of Shc from HER2, and therefore decreases the ERK1/2 phosphorylation in presence of AP150, but not TGFα and heregulin. This suggests that trastuzumab specifically inhibits HER2 homodimer-mediated ERK1/2 phosphorylation, while remaining ineffective for HER2 heterodimer-mediated ERK1/2 phosphorylation. Interestingly, it has been shown that even using the MEK inhibitors which suppresses the ERK1/2 phosphorylation completely does not reduce the viability of trastuzumab-sensitive BC cell lines [24]. This finding indicates that if trastuzumab has any inhibitory role on the MAPK signaling pathway, it will not have significant effects on the proliferation ability of the cells.

## 8. Trastuzumab and PI3K/AKT Signaling Pathway

PI3K/AKT signaling is usually highly activated in various types of cancer [59,60]. This pathway regulates different cellular processes including cell growth, survival, and proliferation [61]. The EGFR signaling pathway activates PI3K/AKT signaling in response to the external stimuli [14]. Among the different members of EGF receptor family, HER3 plays a central role in activating PI3K/AKT signaling by providing several binding sites for p85 subunit of PI3K after being phosphorylated via other HER receptors [17,22,62]. In HER2-positive BC due to the overexpression of HER2, the ligand-dependent or -independent HER2-HER3 heterodimer can be easily formed to activate the PI3K/AKT signaling [24]. Indeed, the HER2-HER3 heterodimer has a more oncogenic role in comparison with the other dimers of HER [52,63]. Generally, upon the binding of the ligand to HER3, these receptors form a heterodimer with other members of the HER family, such as HER2 [19]. The HER3 receptors are phosphorylated by HER2 at different phosphorylation sites to provide several docking sites for the p85 subunit of PI3K [19,20]. After the binding of p85 to HER3, p110 binds to p85 to form the PI3K complex, which then phosphorylates phosphatidylinositol bisphosphate (PIP2) and produces phosphatidylinositol triphosphate (PIP3) [59,64]. AKT proteins bind to PIP3 through its N-terminal pleckstrin homology (PH) domain and are phosphorylated at threonine and serin residues via phosphoinositide-dependent kinase 1 (PDK1) and mammalian target of rapamycin complex (mTORC2), respectively [59]. Phosphorylation of AKT at both threonine and serine amino acids significantly increases its catalytic activity [65]. It is worth mentioning that phosphatase and tensin homolog (PTEN) as a main inhibitor of the PI3K/AKT pathway suppresses AKT phosphorylation by dephosphorylating PIP3, and therefore preventing the association of AKT and PIP3 [66].

Extensive studies have shown that trastuzumab suppresses AKT phosphorylation at both threonine and serine sites [21,23,24,29,52,53,56,57,58,67]. Since HER3 has been well-implicated in the activation of the PI3K/AKT pathway, many groups have studied the effects of trastuzumab on HER2-HER3 heterodimerization and HER3 phosphorylation. As discussed earlier, several studies have demonstrated that trastuzumab suppresses HER3 phosphorylation [24,29,32,52]. On the other hand, our unpublished data, as well as the results published by the others [21,52], have shown that 1 h of trastuzumab treatment in trastuzumab-sensitive BC cell lines does not have any inhibitory effects on HER3 phosphorylation and PI3K activity; however, it drastically reduces the AKT phosphorylation. It seems that this rapid reduction of AKT phosphorylation is through a pathway independent of the PI3K activity and HER2-mediated HER3 phosphorylation. Nagata et al. have found that the short-term treatment of BC cells with trastuzumab reduces the PTEN phosphorylation, and hence induces the PTEN phosphatase activity by increasing the localization of this protein to the cellular membrane [21]. Indeed, patients with PTEN-loss breast tumors are more resistant to trastuzumab compared to BC patients with normal PTEN activity [21]. Interestingly, they also showed that trastuzumab dissociates Src from HER2, and consequently reduces the Src phosphorylation and kinase activity [21]. It has been demonstrated that Src increases the AKT phosphorylation by downregulating the PTEN activity [68]. Taken together, studies suggest that the inhibition of the PI3K/AKT signaling pathway is one of the main mechanisms of action of trastuzumab which is mediated by either the inhibition of HER3 phosphorylation or the activation of PTEN.

## 9. Trastuzumab and Cell Cycle Arrest

The cell cycle consists of four phases, including G0/G1, S, G2, and M phases, which are tightly regulated by several signaling pathways [69]. Complexes of cyclin-dependent kinases (CDKs) and cyclins promote cell cycle progression which are activated and inhibited by mitogenic signals and cell cycle inhibitors, respectively [69]. The cell cycle is usually arrested at the G0 phase and during the G1 phase, cells grow and are prepared to enter the S phase if necessary [70]. Stimulation of the cells with mitogenic stimuli activates signaling pathways regulating CDK4 or CDK6 activation [69]. Generally, the binding of CDK4/6 to Cyclin D mediates the transition of the cell cycle from the G0/G1 phase into the S phase through inhibiting the p21^CIP1^ and p27^KIP1^ function as main inhibitors of CDKs [71]. Moreover, the phosphorylation of the retinoblastoma (RB) protein by the cyclin D-CDK4/6 complex blocks the association of RB and E2F transcription factor, which leads to the activation of E2F-modulated transcription of genes necessary for the entry of the cells into the S phase of the cell cycle, wherein the DNA strands are replicated [71,72].

In the nucleus, p27^KIP1^ binds to the cyclin E-CDK2 complex and represses cell cycle progression from the G1 phase to the S phase [73]. Phosphorylation of p27^KIP1^ at threonine 187 increases its degradation [74]; however, serine 10 phosphorylation stabilizes the protein [75]. Several studies have revealed that trastuzumab arrests the BC cell cycle at the G0/G1 phase through activating the p27^KIP1^ function [30,52,55,76]. Furthermore, Le et al. have shown that trastuzumab induces and reduces the phosphorylation of p27^KIP1^ at serin 10 and threonine 187 phosphorylation sites, respectively [76]. Of note, trastuzumab not only elevates the protein expression of p27^KIP1^ possibly through the inhibition of the protein degradation, but also increases the nuclear localization of this protein in BC cells [52]. Findings suggest that trastuzumab reactivates the p27^KIP1^ function and arrests the cell cycle at the G0/G1 phase via inhibition of AKT phosphorylation [52]. Indeed, AKT mediates the phosphorylation of p27^KIP1^ at threonine 198, which results in the elevation of cytoplasmic localization of p27^KIP1^ [77,78]. The phosphorylation of p27^KIP1^ at this region has been shown to be inhibited upon the treatment of the BC cells with trastuzumab [30]. Moreover, treatment of BT474 BC cells with LY294002, a PI3K inhibitor, induces the localization of p27^KIP1^ into the nucleus and increases the CDK2 association with p27^KIP1^ [52]. Interestingly, trastuzumab downregulates the expression of genes controlling the cell cycle and DNA replication, which their expression can also be downregulated by PI3K inhibitors [67]. Collectively, these results illustrate that the inhibitory effects of trastuzumab on the PI3K/AKT pathway play a critical role in trastuzumab-induced cell cycle arrest.

## 10. Trastuzumab and Antibody-Dependent Cell-Mediated Cytotoxicity (ADCC)

ADCC is one of the main mechanisms of the anti-tumor function of trastuzumab which is mediated by effector immune cells, particularly CD56^dim^CD^16+^ NK cells [79,80,81]. The binding of fragment crystallizable γ receptors (FcγRs) to the Fc region of trastuzumab initiates the ADCC process, which ultimately ends in the secretion of perforin and granzymes from immune effector cells [80,82,83,84]. The binding of FcγR to the Fc portion of the antibody mediates the recruitment of tyrosine-protein kinase Syk to the immunoreceptor tyrosine-based activation motif (ITAM), which activates the SOS/RAS/ERK/p38/MAPK signaling pathway [85,86]. In addition, Syk mediates the generation of phosphatidylinositol 4, 5-bisphosphate [PI(4,5)P2] via the activation of PI3K. Phospholipase C gamma (PLCγ) catalyzes [PI(4,5)P2] into diacylglycerol (DAG) and inositol 1,4,5-trisphosphate (InP3). DAG/PKC induces the MAPK pathway and InP3 mediates the Ca^2+^ release into the cytoplasm from the endoplasmic reticulum (ER) in immune effector cells, which regulates the release of cytoplasmic granules, such as perforin and granzymes [87,88]. Perforin forms pores in the plasma membrane and facilitates granzyme diffusion into the cytoplasm of tumor cells, and granzymes trigger apoptosis in tumor cells by inducing caspase activity and DNA fragmentation [89,90]. Tumor cell lysis increases tumor antigen presentation in the tumor microenvironment and consequently enhances the activation of antigen-presenting cells and the polarization of T cells [91]. Moreover, studies show that trastuzumab also boosts other immune responses against HER2-positive BC cells. Gall et al. have demonstrated that trastuzumab increases HER2 uptake by dendritic cells (DC) when the cells are co-cultured with SKBR3 and BT474 HER2-positive BC cells. Interestingly, they showed that this process is specifically dependent on FcγRs activity of DCs [92].

Tumor cells, regardless of low or high expression levels of HER2, can be coated, albeit to different extents, by antibodies and destroyed eventually in early or late stages of disease [93,94]. However, HER2 overexpression in tumor cell membranes maximizes the ADCC activity [37]. It has been shown that several factors can affect the efficacy of trastuzumab-induced ADCC. For example, the inhibition of EGFR endocytosis, followed by increasing the exposure of the cells to the monoclonal antibody, enhances the ADCC efficacy [95]. Furthermore, low sensitivity to the perforin/granzyme apoptosis pathway makes the tumor cells resistant to the trastuzumab-mediated ADCC [96]. Moreover, single nucleotide polymorphism (SNP) in FcγRs has been suggested to be served as a biomarker in the prediction of BC patients’ response to Fc-mediated ADCC [97,98]. The modification of anti-HER2 monoclonal antibodies’ Fc region to enhance the affinity of the Fc region toward mutant FcγRs can be considered as a treatment strategy to increase trastuzumab-induced ADCC in patients with Fc receptor polymorphisms [99].

## 11. Trastuzumab and HER2 Isoforms

The p95HER2 is an amino terminally truncated membrane-bound fragment which is generated from the cleavage of the extracellular domain (ECD) of full length ~185 kDa HER2. The p95HER2 can be generated through proteinases including A disintegrin and metalloproteinases (ADAM)s and matrix metalloproteinases (MMP)s, or alternative internal translation of HER2 mRNA [100,101]. p95HER2/611 carboxyl-terminal fragments (CTF) (100 to 115-kDa) and p95HER2/648CTF (95 to 100-kDa) are two truncated forms of HER2 which are generated from different translation initiation codons [101]. p95HER2 overexpression is associated with poor prognosis and trastuzumab resistance in patients with aggressive BC [102]. p95HER2, especially p95HER2/611CTF, has been shown to have enhanced homodimerization ability and higher tyrosine kinase activity [103,104,105]. Therefore, p95HER2 overexpression leads to the continuous activation of PI3K/AKT and MAPK signaling pathways in BC cells [103,104,105]. Trastuzumab has been shown to prevent MMP-mediated HER2 shedding, thereby inhibiting p95HER2-regulated downstream signaling pathways like PI3K/AKT [106,107]. However, high expression levels of p95HER2 make the cells resistant to trastuzumab as it cannot bind to p95HER2 due to ECD loss [103]. Parra-Palau et al. showed that the treatment of p95HER2/611CTF-positive breast tumors with doxorubicin makes the tumors sensitive to trastuzumab [108]. Moreover, studies have also focused on using tyrosine kinase inhibitors (TKIs) (e.g., lapatinib) to overcome trastuzumab resistance in BC cells. For example, it has been reported that lapatinib as a dual TKI of HER2 and EGFR inhibits p95HER2 activity and its downstream AKT pathway in BC cells [103]. Heat shock protein 90 (HSP90) overexpression has been shown to contribute to the metastasis of BC cells and poor prognosis in BC patients [109,110]. Interestingly, HSP90 inhibitors destabilize p95HER2 and activate apoptosis in trastuzumab-resistant p95HER2-positive BC cells [111]. It has been demonstrated that other isoforms of HER2 also affect the trastuzumab mode of action. For example, Castiglioni et al. have shown that the deletion of exon 16 of wild-type HER2 produces an HER2 isoform with a shorter ECD [112]. They found that exon-16-deleted HER2 has a lower binding affinity for trastuzumab compared to wild-type HER2 in HEK-293 cells transfected with wild-type and exon-16-deleted HER2 [112]. However, the same group later reported that trastuzumab suppresses the mammary tumor growth and metastasis more efficiently in transgenic mice expressing the exon-16-deleted isoform of human HER2 compared to transgenic mice expressing wild-type human HER2 [113]. In addition, they showed that the expression of the exon-16-deleted isoform of HER2 is positively correlated with Src activation. Interestingly, clinical results demonstrated that trastuzumab significantly decreases the relapse rate in patients with breast tumors expressing high levels of the exon-16-deleted isoform of HER2 and phosphorylated-Src [113]. Palladini et al. have revealed that the expression of wild-type HER2 or exon-16-deleted isoform of HER2 affects the tumor vasculature [114]. They showed that breast tumors expressing wild-type HER2 form few large vessels which negatively impact drug delivery to the tumor. On the other hand, the exon-16-deleted isoform of HER2 induces the formation of many small vessels which facilitates the drug delivery to the tumor and improves the treatment efficiency.

## 12. Mechanisms of Resistance to Trastuzumab

Clinical results show that two-thirds of the patients do not respond to trastuzumab and patients who initially responded subsequently develop resistance to this therapy [115]. To date, several mechanisms have been proposed to explain how BC cells are resistant, or develop resistance, to trastuzumab treatment. Activating mutations in the p110α subunit of PI3K and/or inactivating mutations in PTEN play a crucial role in trastuzumab resistance through persistent activation of the PI3K/AKT signaling pathway [21,116,117]. In addition, some BC cells express truncated HER2, lacking ECD, preventing trastuzumab binding [103,118]. Furthermore, hyperactivation of other tyrosine kinase receptors, such as insulin-like growth factor-I receptor (IGF-IR), compensates for the inhibition of HER2 downstream signaling pathway by trastuzumab [119].

Trastuzumab monotherapy is not the current standard of care, and combinational therapies are administered in attempts to overcome the intrinsic or acquired resistance to trastuzumab. Three different groups of drugs are administered in combinational therapies to increase the efficacy of trastuzumab: (1) trastuzumab-modified or -conjugated drugs. For instance, trastuzumab emtansine (T-DM1) is a conjugated trastuzumab and highly toxic drug derivative of maytansine-1 (DM1) [120]. T-DM1 has been shown to improve clinical outcomes of HER2-positive BC patients [121,122]. (2) Antibodies targeting other domains of HER2 (e.g., pertuzumab) and/or other HER receptors (e.g., patritumab targeting HER3). NEOSPHERE clinical trial results showed that the rate of pathological complete response (pCR) was higher for HER2 positive BC patients treated with a combination of pertuzumab, trastuzumab, and docetaxel compared to patients treated with trastuzumab and docetaxel [123]. (3) TKIs. For example, lapatinib significantly improves the clinical outcomes of combinational therapy of trastuzumab and aromatase inhibitors in HER2-positive BC patients. [124].

## 13. Conclusions and Perspective

**Figure 3:**
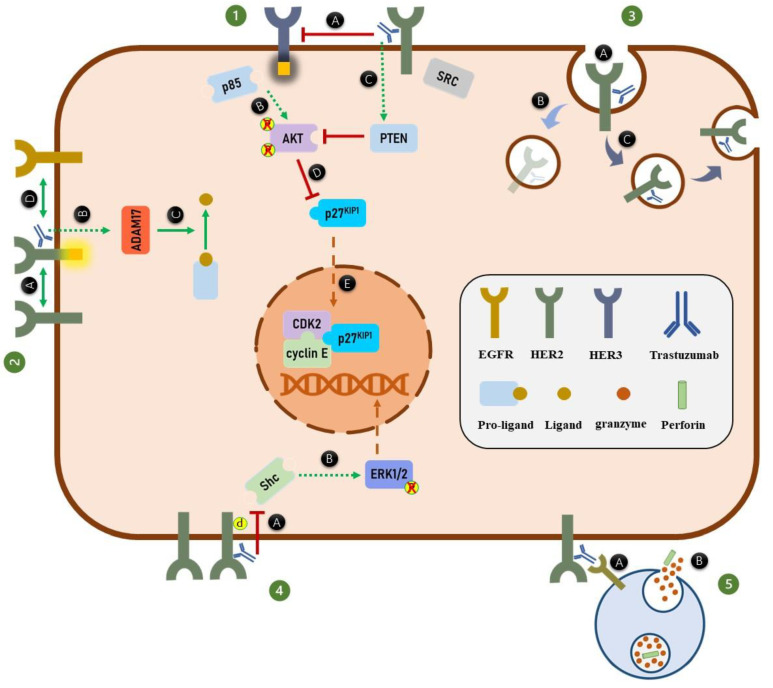
Trastuzumab mechanisms of action. (**1**) Binding of trastuzumab to HER2 receptors inhibits (**A**) the ligand-independent HER2-HER3 heterodimer formation, HER3 phosphorylation, and ultimately association of p85 to HER3 receptors. (**B**) Inhibition of PI3K complex formation inhibits AKT phosphorylation. Moreover, (**C**) trastuzumab inhibits AKT phosphorylation through activation of PTEN phosphatase activity. (**D**) Inhibition of AKT phosphorylation, activates p27^KIP1^ protein. (**E**) The activated p27^KIP1^ enters the nucleus and arrests the cell cycle through suppressing the function of CDK2/cyclin E complex. (**2**) Trastuzumab increases HER2 phosphorylation by (**A**) inducing the HER2 homodimer formation, (**B**) increasing the expression of ADAM17, which (**C**) subsequently increases the HER-specific ligand production. (**D**) Elevation of the HER-specific ligands production from pro-ligands enhances the HER2 heterodimer formation. (**3**) Binding of trastuzumab to HER2 receptors (**A**) induces HER2 internalization. The internalized HER2 can be either (**B**) degraded or (**C**) recycled to the cell membrane. (**4**) Trastuzumab through (**A**) unknown mechanism dissociate Shc from HER2 homodimers which (**B**) ultimately inhibits ERK1/2 phosphorylation. (**5**) Trastuzumab induces the ADCC process. (**A**) Fc region of trastuzumab is recognized by FcγRs of immune effector cells like NK cells. (**B**) Binding of Fc portion of antibody to the FcγRs activates downstream signaling pathways in immune effector cells which results in secretion of perforin and granzymes. The granzymes can induce apoptosis in targeted tumor cells.

In this review, we scrutinized the effects of trastuzumab on HER2 receptors’ functions from HER2 dimerization to HER2-mediated downstream signaling pathways, which are summarized in Figure 3 and Table 1. Generally, four different conditions, including treatment time duration, BC cell types, absence or presence of various HER-specific ligands, and different in vitro and in vivo models affect the mode of action of trastuzumab. This should be considered when interpreting the mechanism of action of trastuzumab. However, regardless of the experimental conditions, the findings strongly suggest that trastuzumab inhibits AKT phosphorylation and arrests the cell cycle through AKT inhibition. Moreover, it is well-documented that trastuzumab significantly activates the ADCC process against the HER2-overexpressed BC tumors. Therefore, the future clinical and experimental studies should focus on the significance of mechanisms through which trastuzumab inhibits AKT phosphorylation, including HER2/HER3/PI3K/AKT and HER2/SRC/PTEN/AKT signaling axes in both resistant and sensitive HER2-positive breast tumors. In addition, further studies on these signaling pathways would help us to have better understanding about the acquired trastuzumab resistance mechanisms. Such studies would benefit us to design novel combination therapies to not only improve the efficacy of trastuzumab, but also overcome the resistance to this drug. Moreover, more studies should be carried out to boost the trastuzumab-activated ADCC process by either modifying the Fc portion of trastuzumab or increasing the activity of human immune effector cells involved in ADCC process.

## Figures and Tables

**Table 1:** The summary of trastuzumab mechanisms of action based on in vitro evidence.

Mechanism of Action	Finding	Experimental Model	Method	Reference
Effect of trastuzumab on HER2 homodimerization	Activatory effect	CHO cells transfected with HER2 receptors	Cross-linking assay	[26]
		BT474 and SKBR3 HER2-positive BC cells	FRET *	[16]
Effect of trastuzumab on ligand-dependent HER2 heterodimerization	No effect	BT474 and SKBR3 HER2-positive BC cells	Co-IP **	[23]
	No effect	SKBR3 HER2-positive BC cells	Co-IP	[24]
Effect of trastuzumab on ligand-independent HER2 heterodimerization	Inhibitory effect	SKBR3 HER2-positive BC cells	Reversible cross-linking followed by Co-IP	[24]
	Inhibitory effect	SKOV3 HER2-positive ovarian cancer cells	TR-FRET ***	[25]
	No effect	BT474 and SKBR3 HER2-positive BC cells	FRET	[16]
Effect of trastuzumab on HER2 phosphorylation	Activatory effect	SKBR3 and BT474 HER2-positive BC cells	WB ****	[29,31,32]
Effect of trastuzumab on HER3 phosphorylation	Inhibitory effect	SKBR3 and BT474 HER2-positive BC cells	WB	[24,29]
Effect of trastuzumab on HER2 endocytosis and downregulation	Activatory effect	SKBR3 HER2-positive BC cells	ICC ^#^ and WB	[34]
	Activatory effect	HER2-positive BC tumor samples	IHC ^##^	[36]
	No effect	SKBR3 HER2-positive BC cells	ICC and WB	[31,38]
Effect of trastuzumab on MAPK signaling pathway	Inhibitory effect	SKBR3 and BT474 HER2-positive BC cells	WB	[52]
	Inhibitory effect	NCI-N87 HER2-positive gastric cell line	WB	[53]
	Inhibitory effect	MCF10A cells transfected with chimeric HER2 and FK506-binding protein (FKBP)	WB	[54]
	Activatory effect	SKBR3 and BT474 HER2-positive BC cells	WB	[29,31]
Effect of trastuzumab on PI3K/AKT signaling pathway	Inhibitory effect	SKBR3 HER2-positive BC cells	ELISA ^###^	[24]
	Inhibitory effect	SKBR3 and BT474 HER2-positive BC cells	WB	[29,52,67]
Effect of trastuzumab on cell cycle	Inhibitory effect	SKBR3 and BT474 HER2-positive BC cells	DNA content quantification using flow cytometry	[30,76]
Effect of trastuzumab on ADCC	Activatory effect	CHO cells transfected with HER2 receptors	Promega ADCC Bioassay kit	[26]
	Activatory effect	SKBR3 HER2-positive BC cells	live-cell imaging	[80]
Effect of trastuzumab on HER2 cleavage	Inhibitory effect	SKBR3 and BT474 HER2-positive BC cells	WB	[107]

* Fluorescence resonance energy transfer, ** Co-immunoprecipitation, *** Time-resolved fluorescence resonance energy transfer, **** Western blot, ^#^ Immunocytochemistry, ^##^ Immunohistochemistry, ^###^ Enzyme-linked immunosorbent assay.
